# Enhanced Electroluminescence from Silicon Quantum Dots Embedded in Silicon Nitride Thin Films Coupled with Gold Nanoparticles in Light Emitting Devices

**DOI:** 10.3390/nano8040182

**Published:** 2018-03-22

**Authors:** Ana Luz Muñoz-Rosas, Arturo Rodríguez-Gómez, Juan Carlos Alonso-Huitrón

**Affiliations:** 1 Centro de Ciencias Aplicadas y Desarrollo Tecnológico, Universidad Nacional Autónoma de Mexico, A.P. 70-180, Ciudad de Mexico 04510, Mexico; analu.mrosas@gmail.com; 2 Instituto de Física, Universidad Nacional Autónoma de Mexico, Circuito de la Investigación Científica s/n, Ciudad Universitaria, A.P. 20-364, Coyoacán, Ciudad de Mexico 04510, Mexico; 3 Instituto de Investigaciones en Materiales, Universidad Nacional Autónoma de México, Ciudad Universitaria, A.P. 70-360, Coyoacán, Ciudad de Mexico 04510, Mexico; alonso@unam.mx

**Keywords:** silicon quantum dots, localized surface plasmon resonances, light emitting devices, gold nanoparticles, electroluminescence enhancement

## Abstract

Nowadays, the use of plasmonic metal layers to improve the photonic emission characteristics of several semiconductor quantum dots is a booming tool. In this work, we report the use of silicon quantum dots (SiQDs) embedded in a silicon nitride thin film coupled with an ultra-thin gold film (AuNPs) to fabricate light emitting devices. We used the remote plasma enhanced chemical vapor deposition technique (RPECVD) in order to grow two types of silicon nitride thin films. One with an almost stoichiometric composition, acting as non-radiative spacer; the other one, with a silicon excess in its chemical composition, which causes the formation of silicon quantum dots imbibed in the silicon nitride thin film. The ultra-thin gold film was deposited by the direct current (DC)-sputtering technique, and an aluminum doped zinc oxide thin film (AZO) which was deposited by means of ultrasonic spray pyrolysis, plays the role of the ohmic metal-like electrode. We found that there is a maximum electroluminescence (EL) enhancement when the appropriate AuNPs-spacer-SiQDs configuration is used. This EL is achieved at a moderate turn-on voltage of 11 V, and the EL enhancement is around four times bigger than the photoluminescence (PL) enhancement of the same AuNPs-spacer-SiQDs configuration. From our experimental results, we surmise that EL enhancement may indeed be due to a plasmonic coupling. This kind of silicon-based LEDs has the potential for technology transfer.

## 1. Introduction

Since the first reports of luminescence and electroluminescence, originated by quantum size effects, from highly confined silicon materials (superlattices, quantum dots, and quantum wires) [1,2,3], there has been a growing interest in the development of monolithic silicon photonics as the optical analogue of silicon microelectronics [4,5,6,7]. In order to meet this goal, arduous work has been done over the years to fabricate light emitters and electroluminescent devices based mainly on crystalline and amorphous silicon quantum dots (SiQDs) embedded in silicon nitride and silicon dioxide films, and to tune the photoluminescence by controlling the size and the surface passivation of the SiQDs [8,9,10,11,12,13]. However, the silicon photonics have evolved slowly, mainly because the illumination efficiency from confined silicon is still very low compared with that of the direct-band gap III–V semiconductors [7]. 

With the purpose of increasing the efficiency and controlling the light emission from SiQDs, several research groups have used different configurations of noble metals (Au, Ag) nanoparticles and nanostructures in the vicinity of silicon quantum dots. One of the first pioneer works, reported local field-enhanced light emission from silicon nanocrystals implanted in quartz close to a surface film of nanoporous gold, prepared by wet chemical methods [14]. In other posterior works, authors have reported enhanced luminescence from SiQDs implanted in quartz and coupled to Ag island arrays fabricated by electron beam lithography or by subsequent implantation. Those enhancements are reported at emission frequencies that correspond to the collective dipole plasmon resonances [15,16,17,18]. In this regard, we recently reported photoluminescence (PL) enhancement from thin films of SiQDs embedded in silicon nitride coupled to a monolayer of Au nanoparticles which are separated by a nanometric dielectric silicon nitride thin film [19]. In that article, the films were deposited using dry and low-temperature techniques highly compatible with the pre-existing silicon microelectronics technology, such as remote plasma enhanced chemical vapor deposition (RPECVD) and direct current (DC) sputtering. 

A shared feature in the coupled structures reported in all these previous works is that all of them have a spacer between the metal and the SiQDs. In turn, the spacer must be a dielectric, and must have a well-defined thickness ranging from 10 to 20 nm; when these two last conditions are not met, then the metal-spacer-SiQDs structure, far from improving its PL, decreases it [17]. All these works also shared the proposal that the PL enhancement was due to effects of localized surface plasmon resonance (LSPR) [16,17,18,20]. Therefore, they have motivated the development of other plasmonic coupled systems with enhanced PL, such as core-shell-type SiQDs-based nanocomposites consisting of a Au nanoparticle (NP) core and a thick shell of SiQDs agglomerates [21], a structure where SiQDs are placed in a gap between a gold thin film and an Au nanoparticle [22]. Or finally, a structure composed of a monolayer of luminescent SiQDs and a silver (Ag) film over nanosphere (AgFON) plasmonic structure, separated with a polymer spacer [23]. 

The same approach of coupling SiQDs to localized surface plasmons (LSP) has also been applied to enhance the electroluminescence of electroluminescent devices based on SiQDs embedded in silicon-rich silicon nitride (SiN*_x_*(SiQDs)) films deposited by plasma enhanced chemical vapor deposition (PECVD) [24,25,26]. In these works, the enhancement of the electroluminescence by LSP was investigated in two different types of light emitting devices and/or diodes (LEDs) with layered structures, such as ITO-Ni-Au(transparent electrode)/SiN_x_(SiQDs)/Ag islands/p/p^+^-Si(substrate)/Ni-Au(back electrode) [24], ITO/SiO_2_/SiN_x_(SiQDs)/Ag islands/p/p^+^-Si/Al [25,26]. In both types of layered LEDs, the large enhancement of the electroluminescence (EL) (up to 434% relative to SiQD LED without an Ag layer) was attributed to the Ag island layer, which gives rise to an increase in the radiative efficiency as a result of SiQDs-LSP coupling. Additionally, it was also observed, an increase in the current injection efficiency through improved carrier tunneling between the rough surface of the Ag layer and the SiQDs. It is also worth mentioning that in the case of the second type of layered LEDs, the effect of the SiO_2_ layer was not discussed. 

An interesting work that questions the mechanisms of the enhancement of the spontaneous emission rate due to the LSP coupling between the SiQDs excitons and the Ag island layer, is the one authored by Baek Kim and collaborators. There, they report an enhancement of 493% in the EL of a LED with a layered structure NiO-Ni-Au(transparent electrode)/SiN*_x_*(SiQDs)/rough p^+^-Si, which do not contain any Ag layer, but instead, it was fabricated on a nano-roughened Si substrate [27]. In a recent work, novel electroluminescent structures were fabricated using SiQDs/SiO_2_ multilayers fabricated by PECVD on a Pt nanoparticle—sputtering-coated Si nanopillar array substrate, with ITO as the transparent electrode. The electroluminescence enhancement observed in these EL structures was attributed to both, the possible resonance coupling between the localized surface plasmon (LSP) of Pt NPs, and the band-gap emission of SiQDs/SiO_2_ multilayers, and the surface roughening originated by the nanopillar array [28].

In this work, we have investigated the electroluminescence of four different configurations of metal insulator semiconductor (MIS)-type nano-layered structures using SiQDs embedded in silicon nitride luminescent films and dielectric silicon nitride (as spacer), both deposited by remote PECVD (RPECVD), and gold nanoparticles (AuNPs) deposited by sputtering on p-type silicon substrate. We found that there is a maximum EL enhancement when the appropriate AuNPs-spacer-SiQDs configuration is used. Furthermore, it was identified that the EL enhancement is around four times bigger than the PL enhancement previously observed in an identical nano-layered configuration [19]. From this work, we can conclude that the EL enhancement may indeed be due to a plasmonic coupling. Nevertheless, we also identify that the presence of gold nanoparticles in the EL device allow a more efficient distribution of charge carriers towards the luminescent centers (SiQDs). Consequently, we confirm that more than one mechanism could be involved in the optimized electroluminescence. 

## 2. Methods

To investigate the effect of gold nanoparticles in the vicinity of silicon quantum dots on the electroluminescence of the fabricated light emitting devices (LED), we used p-type silicon wafers (100), with a concentration of 10 × 10^15^ holes, as the semiconductor substrate. Common solvent cleaning was employed for all samples, and additionally a standard cleaning 1 as the widely reported by Radio Corporation of America (RCA clean) was used for silicon wafers. Gold nanoparticles (AuNPs) were deposited using a Cressington 108 Sputter Coater (TED PELLA, INC., Redding, CA, USA) in 0.8 mb argon atmosphere. Quartz substrates were selectively used to make depositions of the different films in order to measure their absorption spectrum.

Silicon nitride films with different thicknesses and compositions were deposited using a remote plasma enhanced chemical vapor deposition (RPECVD) system whose characteristics have been reported elsewhere [29]. A substrate temperature of 300 °C, radio frequency power of 150 watts and pressure of the reaction chamber of 300 mT were used as deposition parameters. The flow rates of H_2_, Ar, and SiH_2_Cl_2_ were 10, 75, and 5 sccm, respectively for all the deposited films. A NH_3_ flow rate of 600 sccm was settled to attain a non-radiative silicon nitride (SiN*_x_*) insulating film with NH_3_/SiH_2_Cl_2_ gas flow ratio of *R* = 120. In addition, a NH_3_ flow rate of 200 sccm was used to obtain the radiative silicon rich silicon nitride film (SiQDs) with a NH_3_/SiH_2_Cl_2_ gas flow ratio of *R* = 40. 

**Figure 1:**
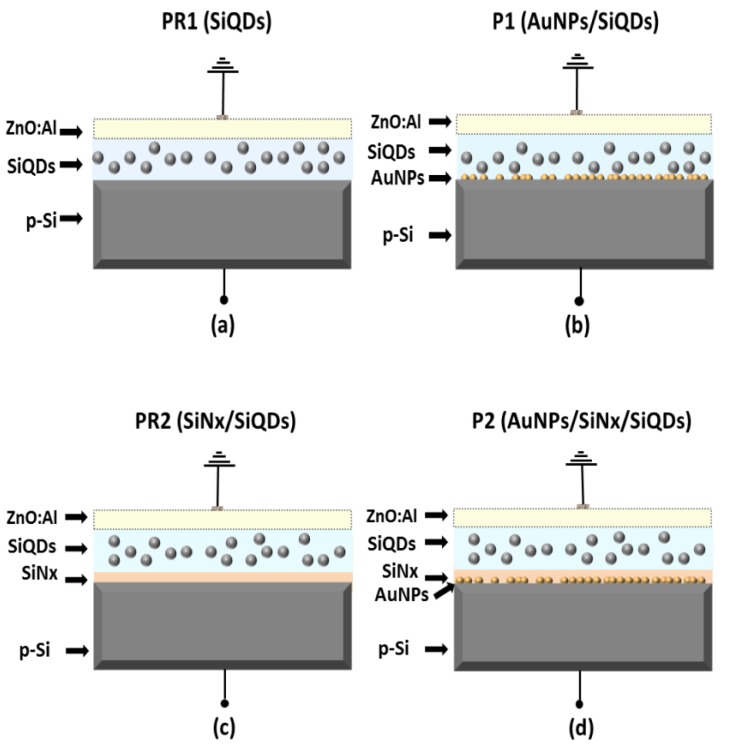
(**a**–**d**) Schematic representation of the fabricated structures PR1, P1, PR2, and P2.

Four different types of LED structures were fabricated with the aim of obtaining separate information on the role of the AuNPs and silicon nitride layers in the enhancement of the electroluminescence of the devices. Two EL structures were fabricated without AuNPs, and these were considered as reference structures. The first reference EL structure was fabricated by depositing an 80 ± 5 nm thick silicon-rich silicon nitride (SiQDs) on the surface of the p-type silicon substrate, and then a ZnO-Al film, resulting in the (p-Si/SiQDs (80 ± 5 nm)/ZnO-Al) structure, named PR1 (see Figure 1a). The second reference structure was fabricated as the previous PR1 structure, but depositing before the SiQDs film a 10 ± 2 nm thick non-radiative silicon nitride (SiN*_x_*) film. This (p-Si/SiN*_x_* (10 ± 2 nm)/SiQDs (80 ± 5 nm)/ZnO-Al) reference structure was named PR2 (see Figure 1c). On the other hand, the first AuNPs-enhanced electroluminescent structure was fabricated also as the PR1 structure but depositing a layer of AuNPs before the SiQDs film as shown in Figure 1b). This (p-Si/AuNPs/ SiQDs (80 ± 5 nm)/ZnO-Al) structure was named P1. The second AuNP-enhanced electroluminescent structure was fabricated as the PR2 structure, but depositing a layer of AuNPs before the non-radiative silicon nitride (SiN*_x_*) film. This (p-Si/AuNPs/SiN*_x_* (10 ± 2 nm)/SiQDs (80 ± 5 nm)/ZnO-Al) was named P2 (see Figure 1d). The different silicon nitride layers were grown into the same chamber without exposition of the films to the ambient atmosphere by changing the NH_3_ gas flow rate. As transparent conductive contact (TCC), aluminum doped zinc oxide (ZnO-Al) was deposited by ultrasonic spray pyrolysis on top of the silicon-rich silicon nitride layer, defining square patterns with sides of 2 mm. Finally, an aluminum metal layer of 100 nm was used as the bottom electrode, deposited by vacuum evaporation for all the devices.

The presence of the SiQDs and Au nanoparticles were characterized by high-resolution transmission electron microscopy (HRTEM) using a field emission gun JEM-2010F microscope (JEOL, INC., Peabody, MA, USA) operating at 200 kV. The HRTEM images were digitally treated with a GATAN micrograph system (GATAN, INC., Pleasanton, CA, USA). The thickness (Th) of the films was measured using a manual Gaertner 117 ellipsometer (Gaertner Scientific Coroporation, Chicago, IL, USA) equipped with a He-Ne laser (632 nm). Ultraviolet-visible (UV-vis) transmission measurements were carried out in the range from 300 to 1100 nm using a double-beam PerkinElmer Lambda 35 UV-vis spectrophotometer (PerkinElmer, Billerica, MA, USA). The chemical composition of the layers was determined by Fourier transform infrared spectroscopy (FTIR) by using a Nicolet 6700 system (Nicolet Instrument Corporation, Madison, WI, USA). X-ray photoelectron spectroscopy (XPS) depth profiles were performed in an ultra-high vacuum system scanning XPS microprobe PHI 5000 VersaProbe II (PHYSICAL ELECTRONICS, Inc., Chanhassen, MN, USA). Photoluminescence (PL) and electroluminescent (EL) measurements were carried out in a dark room at room temperature. PL spectra were obtained using an unfocused beam of 25 mW from a Kimmon He-Cd laser operating at 325 nm (3.81 eV) (Kimmon Electric US, Ltd., Englewood, CO, USA). An 2200-72-1 power supply (Keithley Instrumemts, Cleveland, OH, USA) source and Digital Multimeter Tektronix DMM4050 (Tektronix, Beaverton, OR, USA) were used as power source and current meter, respectively. The PL and EL spectra were recorded with a Fluoromax-Spex spectrofluorometer (SPEX Industries, Edison, NJ, USA) at room temperature. Finally, a field emission-scanning electron microscope (JEOL7600F FE-SEM) (JEOL, Inc., Peabody, MA, USA) was used to observe the cross-section of the Metal-Insulator-Semiconductor (MIS-type devices). 

## 3. Results and Discussion

### 3.1. Preparation of the Layered Luminescent Devices

To characterize the chemical and physical properties of the SiQDs and SiN*_x_* films used for the fabrication of the devices, similar thicknesses of each these layers were deposited on silicon and quartz (Table 1). From Tauc plots [30], the band gap of the SiQDs and SiN*_x_* films were obtained and are presented in Table 1, which are 4.04 and 4.68 eV, respectively. As expected, the SiN*_x_* layer with *R* = 120 has a wider band gap than the SiQDs layer with *R* = 40, and closer to that of the stoichiometric Si_3_N_4_ (5 eV) [31,32]. The latter is in agreement with those works which have demonstrated that a higher NH_3_ flow rate, as one of the precursors for the deposition of silicon nitride films, increases its band-gap energy, since Si atoms are bonded to more N atoms, due to the larger electronegativity of N compared to that of Si and H [33]. Then, the gap energy of the SiQDs layer should be lower due to its higher content of silicon, whose band gap is 1.1 eV [31,32]. Furthermore, the refractive index of the SiQDs film obtained by null ellipsometry was slightly higher than that of the SiN*_x_* layer, owing to its higher silicon content. However, it is worth noting the low value of the refractive index of the SiN*_x_* film compared to the stoichiometric Si_3_N_4_ value of 2. A similar trend of the refractive index has been observed in silicon nitride films when increasing the flow rate of the NH_3_ precursor, probably due to an increase in the H and N content into the films [34,35]. 

**Figure 2:**
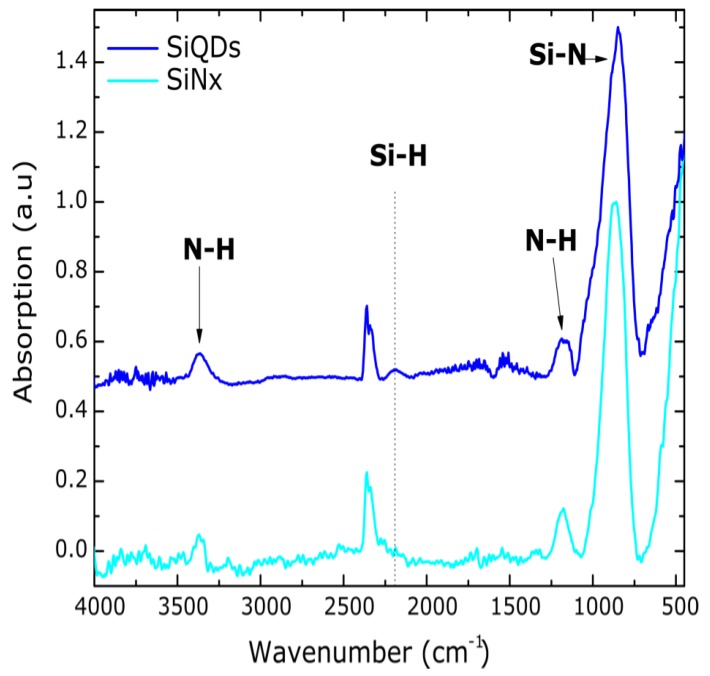
FTIR spectra for the two different NH_3_ flow rates used to attain silicon quantum dots (SiQDs) and non-radiative silicon nitride (SiN*_x_*) films. The band at 2360 cm^−1^ corresponds to the CO_2_ molecule in the operating environment.

The Fourier transform infrared spectra of the SiQDs and SiN*_x_* films are depicted in Figure 2. In both samples, the characteristic Si–N (840 cm^−1^), N–H (1180 cm^−1^), and N–H (3350 cm^−1^) bands of silicon nitride are found. However, the Si–H (2190 cm^−1^) band is only clearly observed in the SiQDs layer. The lack of this band in the SiN*_x_* film with a higher NH_3_ flow rate should be due to the incorporation of nitrogen atoms in silicon sites of the Si–H groups, which is consistent with those works using a wide range of this precursor gas [33,35].

**Figure 3:**
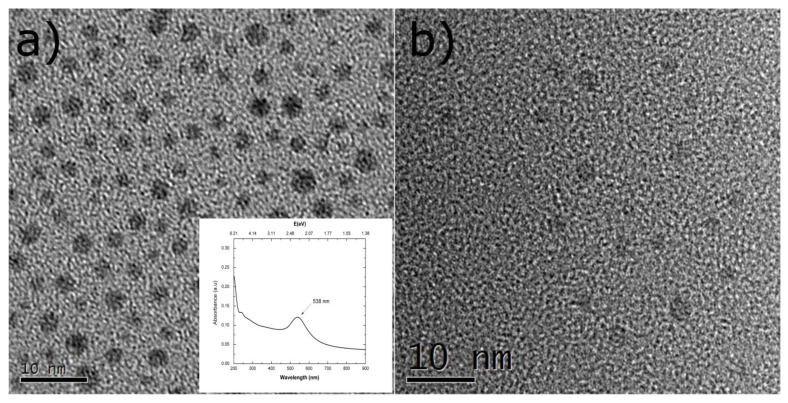
HRTEM images of gold nanoparticles (AuNPs) (**a**) and SiQDs (**b**) films. The average size and superficial density of AuNPs were 2.9 nm and 2.52 × 10^12^ particles/cm^2^, respectively; meanwhile, for silicon nanoparticles, they were 3.1 nm and 6.04 × 10^12^ particles/cm^2^, respectively [11,19]. The cover surface of gold nanoparticles was 18.12%, and its plasmonic resonance location was found at 538 nm (inset of Figure 3a).

The gold nanoparticles (AuNPs) and silicon quantum dots (SiQDs) thin films were obtained using deposition conditions previously studied, which gave rise to average particle sizes of 2.9 nm and 3.1 nm, respectively [11,19]. It can be observed in both samples from HRTEM images (Figure 3a,b) a uniform distribution of particles throughout the whole surface and quasi-spherical shape. Likewise, the cover surface of gold nanoparticles obtained by HRTEM micrographs was 18.12% and its plasmonic resonance location was found at about 538 nm (inset of Figure 2a).

**Figure 4:**
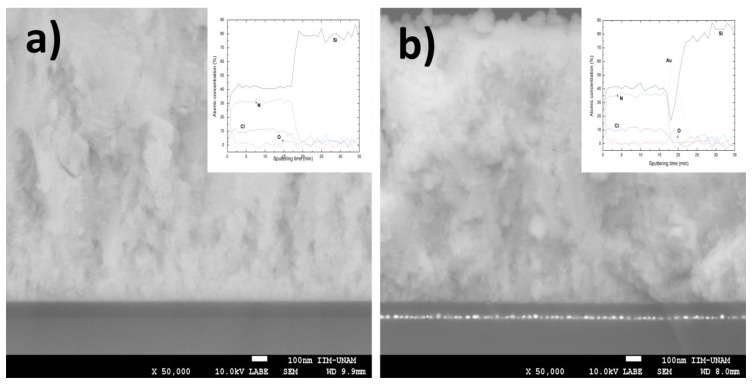
Cross-sectional views by SEM of the (**a**) PR2 (p-Si/SiN*_x_*/SiQDs/ZnO-Al) and (**b**) P2 (p-Si/AuNPs/SiN*_x_*/SiQDs/ZnO-Al) structures, respectively. The different silicon nitride layers (SiN*_x_* and SiQDs) are not distinguishable by this microscopy technique. The depth profiles of these samples from top silicon nitride to silicon substrate are inset of each figure.

Cross-sectional SEM images of the PR2 (p-Si/SiN*_x_*/SiQDs) and P2 (p-Si/AuNPs/SiN*_x_*/SiQDs) structures are depicted in Figure 4a,b, respectively. From these images, the ZnO-Al transparent conductive contact can be identified at the top of the structures. It is worth noting that the SiN*_x_* and SiQDs layers are not distinguishable by this technique, in both samples and that the AuNPs in the P2 structure are clearly located at the interface between the silicon nitride and the silicon substrate. The XPS depth profiles of the PR2 and P2 samples from silicon nitride to silicon substrate are the inset of Figure 4a,b, respectively. These profiles show atomic concentrations of Si 2p, N 1s, and Cl 2p in the silicon nitride films, as well as no diffusion of them to the silicon substrate. Additionally, it is possible to identify that Au atoms do not diffuse to the bulk of the SiQDs layer. A content of oxygen is observed at the surface of these samples, which has been attributed to post-deposition reactions, occurred when the films were exposed to ambience [36,37].

### 3.2. Electroluminescence

**Figure 5:**
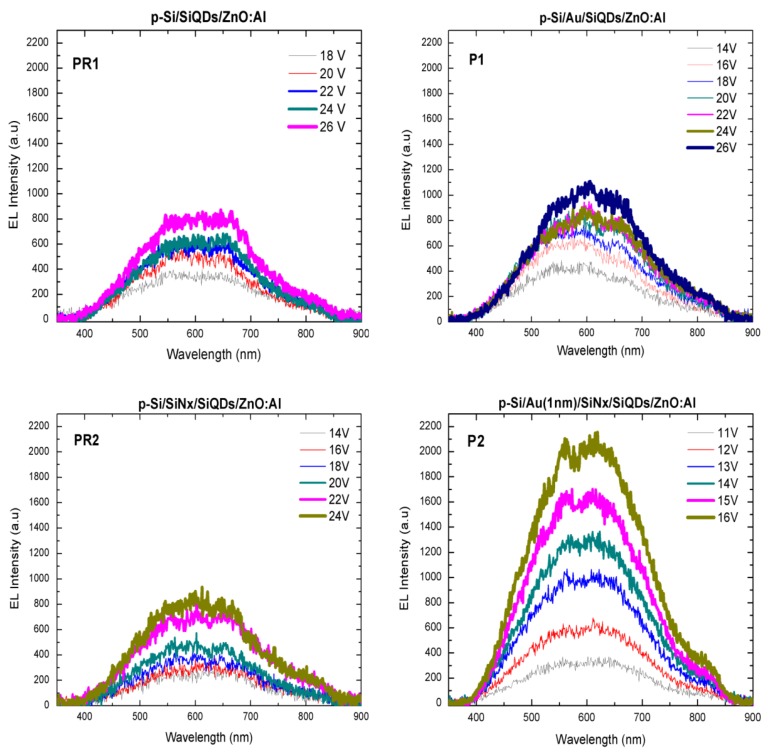
Electroluminescent (EL) spectra of the PR1 (p-Si/SiQDs/ZnO-Al), P1 (p-Si/AuNPs/SiQDs/ZnO-Al), PR2 (p-Si/SiN*_x_*/SiQDs/ZnO-Al) and P2 (p-Si/AuNPs/SiN*_x_*/SiQDs/ZnO-Al) samples under forward bias.

The electroluminescence spectra of the four structures were obtained only in forward bias (considered when the cathode electrode is on the ZnO-Al) when applying voltages greater than 10 V, as can be seen from Figure 5. From this image, all the structures show an increment of the electroluminescent emission with increasing voltage at room temperature and a maximum intensity peak centered at around 600 nm. Also, it can be observed the influence of gold nanoparticles on the EL turn-on voltages of the fabricated structures, since for the PR1 and P1 samples (without the SiN*_x_* layer), the EL turn-on voltages are 18 V and 14 V, respectively, i.e., lower for the sample with AuNPs. For these samples, voltage steps of 2 V are required to observe increased emission intensity. Likewise, the EL turn-on voltage is also lower for the P2 sample (11 V) with gold nanoparticles when compared with the reference PR2 (14 V) sample without them. Increased EL intensity is obtained using voltage steps of 1 V and 2 V for these samples, respectively.

**Figure 6:**
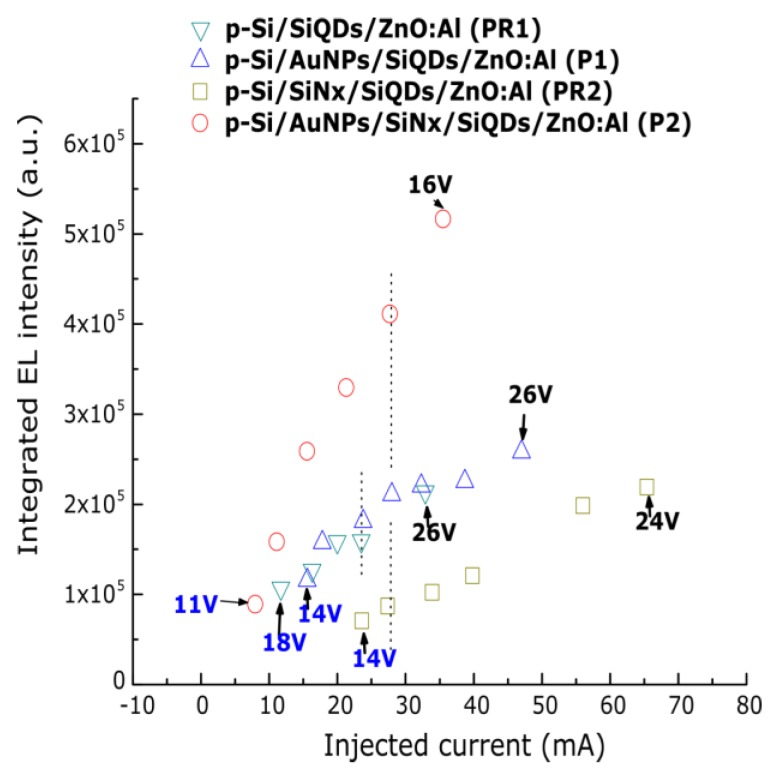
Integrated EL intensity vs injected current (mA) of the samples P1, PR1, P2, and PR2. A maximum EL enhancement (considered as the ratio of integrated EL intensity of devices with gold nanoparticles and reference devices) of 4.7 at ~27.7 mA is found for the P2 sample when compared to the reference PR2 sample.

The integrated electroluminescence intensity against the injected current of each sample is depicted in Figure 6. At first glance, a similar trend is observed for the P1 and PR1 samples; however, a slight EL enhancement (considered as the ratio of integrated EL intensity of devices with gold nanoparticles and reference devices) of 1.14 is found for these samples at about the same current of 23.5 mA. Moreover, the current injection is higher in the P1 sample when compared to the PR1 sample at the same applied voltage. The structures with the thin SiN*_x_* layer (P2 and PR2) show an EL enhancement of 4.7 at ~27.7 mA. For these samples, the current injection is also higher for the sample with gold nanoparticles from 15 V. These results suggest an improvement of the external quantum efficiency in those samples using AuNPs. 

In our previous paper, the PL emission of the four structures fabricated in this work had a Gaussian-like shape with a broad band centered at about 505 nm [19], manly attributed to quantum confinement effect in SiQDs. Since their corresponding EL emissions peaks were red shifted at ~600 nm, it is difficult to elucidate if the origin of EL is the same as that of PL, as other mechanisms with lower radiative transition probability as defects in the matrix or interface states in SiQDs (which produce radiative events at higher wavelengths) [27,38,39] could give rise to the observed EL emission.

**Figure 7:**
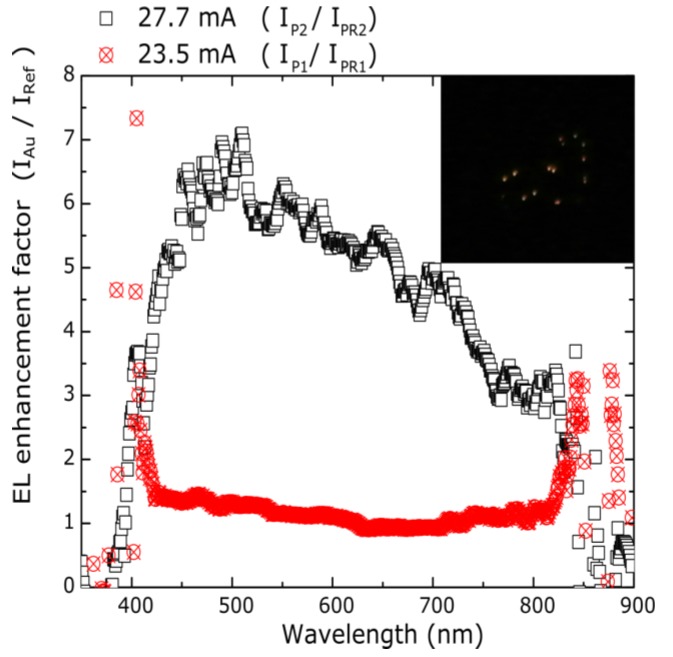
EL enhancement factor (defined as the ratio of EL intensities of samples with gold nanoparticles and their references ones (IP1(λ)/IPR1(λ) and IP2(λ)/IPR2(λ)). The IP1(λ)/IPR1(λ) ratio evaluated through a wavelength range from 430 to 815 nm at 23.5 mA, an almost constant line slightly above one is observed. The IP2(λ)/IPR2(λ) ratio at 27.7 mA shows a maximum EL enhancement factor of 7 at about 510 nm.

The EL spectral enhancement factor, defined as the ratio of EL intensities of samples with gold nanoparticles and their references ones (IP1(λ)/IPR1(λ) and IP2(λ)/IPR2(λ)) is shown in Figure 7 for a defined injected current. For the IP1/IPR1 ratio at 23.5 mA, an almost constant line slightly above one is observed with some peaks at the beginning and at the end of the plot, probably due to the noise interfering with the measured signal at low emission intensities. This enhancement factor evaluated through a wavelength range from 430 to 815 nm has a maximum value of 1.45 at about 466 nm, which could indicate that the presence of gold nanoparticles could help to distribute carriers to the luminescent centers of the SiQDs film more efficiently in the P1 sample. On the other hand, the IP2/IPR2 ratio at 27.7 mA through the same wavelength range shows a maximum EL enhancement factor of 7 at about 510 nm, and 5.4 at about the maximum EL intensity peak (~600 nm). It is worth noting that the higher EL enhancement factor in these structures is close to the absorption peak of gold nanoparticles at 538 nm (inset of Figure 2a), which could suggest a resonant coupling between the silicon quantum dots emission and the oscillations in noble metal nanoparticles in the P2 sample [24]. Moreover, as the maximum IP1/IPR1 ratio was found to be lower than the maximum IP2/IPR2 ratio, in spite of the presence of metal nanoparticles in the P1 sample, it is important to take into account the role of the thin silicon nitride layer (SiN*_x_*) separating the noble metal nanoparticles and the SiQDs film in the PR2 and P2 structures, which we will discuss later in this paper. The second factor of 5.4 is close to the EL enhancement of 4.7 earlier observed. Additionally, according to some authors [27,38,39,40,41], we observe shining spots on the top area of our devices, increasing in number as the current increased. Morales-Sánchez and Cabañas Tay et al. [40,41] explain the presence of these shining dots due to the formation of current paths connecting the top and bottom electrodes in silicon-rich oxide-based devices. Considering this, charge carriers under forward bias in our structures would create these paths hindered by the barriers of the bulk matrix and produced during their transport radiative and non-radiative transitions. An image of the luminescent dots obtained in the PR2 sample at 0.69 A/cm^2^ is shown inset of Figure 7.

### 3.3. J-E Characteristics

**Figure 8:**
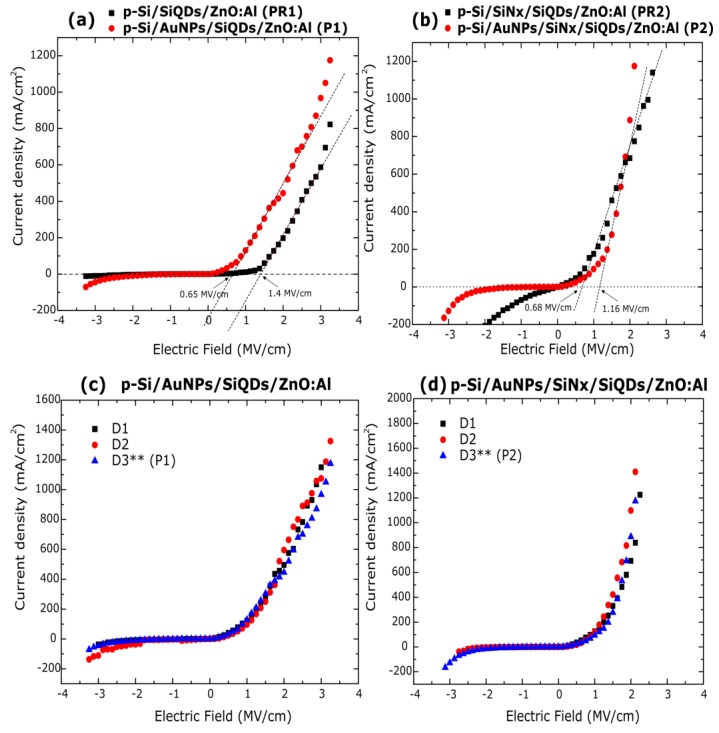
Current density-electric field characteristics of the (**a**) P1 and PR1 and (**b**) P2 and PR2 samples. Current density-electric field plots of three different devices of the P1 (**c**) and P2 (**d**) structures; a double asterisk denotes the P1 and P2 samples in each graph corresponding to the device D3.

Current density vs electric field (J–E) plots of the four structures studied in this work are depicted in Figure 8a,b. The P1 and PR1 samples have threshold electric fields of ~0.65 MV/cm and ~1.4 MV/cm, respectively. Just after current conduction is established, very similar slopes are observed in these structures (Figure 8a). It is possible to observe a lower threshold voltage in the P1 sample; this could be due to increased injection of carriers by the presence of gold nanoparticles. In addition, it is possible that the electrons from the gate could be drifted to the lower interface of the structure by areas of a higher electric field. Once there, the electrons can tunnel to the silicon nitride matrix, and to a lesser extent, to the SiQDs. The latter is possible in our device, since it has been studied that rms roughness as low as some nanometers at the bottom interface of a capacitor can be related to the increased leakage current through it, due to the enhanced local electric field at the protrusions [42,43]. Even though the morphology of gold nanoparticles could not be clearly detected by the AFM (Atomic Force Microscopy) technique, its average diameter size (estimated by TEM images of ~2.9 nm) suggests that this mechanism could be responsible for the lower threshold voltage and higher injection current at the same applied electric field in the P1 sample than PR1 sample. We could expect this effect to be less important at the top interface of the device, as these metal nanoparticles were not shown to increase, significantly, the surface roughness of the SiQDs layer with regard to the SiQDs layer of the reference device. Additionally, from previous results [19], no PL enhancement nor quenching of the P1 and PR1 structures was found, and we could indirectly assume that the reflectance due to the AuNPs is inappreciable. Therefore, the slight EL enhancement observed in these structures at about the same injected current should be due to a more efficient carrier injection to the luminescent centers of the active film in the P1 sample, and as a result, a higher number of conductive paths. 

P2 and PR2 samples have threshold electric fields of ~1.16 MV/cm and ~0.68 MV/cm, respectively, i.e., lower for the reference structure (Figure 8b). For the PR2 sample, it is possible to observe current density under reverse bias, and higher forward current injection than in P2 sample, from low electric fields up to 1.75 MV/cm, after which the current increases faster in the P2 sample under a larger bias condition. The characteristics found in the PR2 sample could have their origin in the intrinsic properties of the silicon nitride films with different chemical composition. The presence of the very thin SiN*_x_* layer (10 nm) with higher band gap than the SiQDs layer (according to the Tauc plots obtained) could increase the number of accumulated holes under forward bias in the p-Si surface at the bottom interface, and give rise to abrupt injection of carriers from the silicon substrate by tunneling towards the SiQDs layer. Moreover, the low refractive index of the SiN*_x_* film indicates a high number of voids in the film, which would promote leakage current even at low electric fields. Since the integrated EL intensity in the PR2 sample was the lowest observed in the fabricated set of samples, we could deduce an inefficient carrier transport to the luminescent centers in the bulk of the active film, in spite of higher current passing through it from low electric fields. 

On the other hand, the higher threshold voltage in the P2 sample may be due to a screening effect of the applied electric field by gold nanoparticles at the bottom interface of the structure [44]. This effect is not seen in the P1 sample (p-Si/AuNPs/SiQDs/ZnO-Al) where the electric field is higher, in a range from 0.74 MV/cm up to 1.49 MV/cm, than in P2 sample. One possible explanation is that silicon nanoparticles growing from the substrate in the P1 structure may work as conduction points of carriers and interact with the previously deposited AuNPs distributed throughout the substrate surface, making the p-Si/AuNPs/SiQDs interface inhomogeneous. 

This direct interaction between silicon and gold nanoparticles is avoided by the low silicon content SiN*_x_* layer in the P2 sample. Therefore, the layered configuration of the P2 sample, as a whole, should be responsible for its higher integrated EL intensity when compared with any of the other fabricated structures. The screening effect of the electric field observed in this sample could promote overlapping of the electron and hole population in regions far from the surface of the SiQDs film where the radiative recombination generally occurs [45]. Likewise, an almost five-fold enhancement of integrated EL intensity and the maximum EL enhancement factor IP2/IPR2 at about 510 nm could suggest an increment of the internal quantum efficiency by coupling of the local field near the surface of metal nanoparticles, and emission of the active layer at a distance defined by the SiN*_x_* thickness (10 nm). 

Since the integrated EL enhancement is larger than the integrated PL enhancement (~2) in the P2 sample [19] with regard to its reference one, it is possible that more than one mechanism is involved in the increase of electroluminescence observed, though a more detailed study is required to throw more light on the issue. Additionally, it was observed high reproducibility of the electrical characteristics of samples with gold nanoparticles, which is shown for three different devices (named D1, D2, D3), using the P1 and P2 configurations in Figure 8c,d, respectively. A double asterisk denotes the P1 and P2 samples in each figure labeled as the D3 device. The good reproducibility of the electrical and optical properties of these structures makes it possible its use in high-reliability applications.

## 4. Conclusions

We fabricated light emitting devices based on silicon nitride films layered structures that explored the role of AuNPs in the vicinity of a SiQDs active film. We observed improved carrier injection in samples using gold nanoparticles under determined bias condition, as well as lower electroluminescent (EL) turn-on voltages. Also, an EL enhancement was found in these samples when compared with their reference ones without noble metal particles, being higher for that sample using a non-radiative SiN*_x_* layer of 10 nm between the AuNPs and the SiQDs film (P2 sample). The almost five times higher integrated electroluminescence observed in this sample can be explained by considering the intrinsic physical properties of the different layers of nitride and metal that make up the device, such as band gap and roughness. However, it is also possible that the observed EL enhanced emission in this multilayer sample could be originated by the plasmonic coupling between AuNPs and the radiative SiQDs film.

## Figures and Tables

**Table 1:** Some physical properties of the silicon nitride layers with different chemical composition.

*R* = NH_3_/SiH_2_Cl_2_ Gas Flow Ratio	Sample	Thickness (nm)	Refractive Index	Optical Band Gap (eV)
120	SiN*_x_*	96.5	1.78	4.68
40	SiQDs	97.9	1.84	4.04
